# Metabolomic Profiling and Functional Characterization of Biochar from Vine Pruning Residues for Applications in Animal Feed

**DOI:** 10.3390/ani14233440

**Published:** 2024-11-28

**Authors:** Serena Reggi, Sara Frazzini, Maria Claudia Torresani, Marianna Guagliano, Cinzia Cristiani, Salvatore Roberto Pilu, Martina Ghidoli, Luciana Rossi

**Affiliations:** 1 Department of Veterinary Medicine and Animal Sciences—DIVAS, University of Milano, 26900 Lodi, Italy; sara.frazzini@unimi.it (S.F.); luciana.rossi@unimi.it (L.R.); 2 Biotecnologie BT srl, PTP, 26900 Lodi, Italy; ctorresani@biotecnologiebt.it; 3 Department of Chemistry, Materials and Chemical Engineering—Giulio Natta, Politecnico of Milan, 20133 Milano, Italy; marianna.guagliano@polimi.it (M.G.); cinzia.cristiani@polimi.it (C.C.); 4 Department of Agricultural and Environmental Sciences—Production Landscape and Agroenergy, University of Milano, 20133 Milano, Italy; salvatore.pilu@unimi.it (S.R.P.); martina.ghidoli@unimi.it (M.G.)

**Keywords:** biochar, vine biomass, circular economy, sustainability, functional properties, feed

## Abstract

Biochar has gained interest as a feed ingredient in livestock nutrition due to its functional properties, circularity, potential to reduce environmental impact, and alignment with sustainable agro-zootechnical practices. The in vivo effects of biochar are closely tied to its physical characteristics, which vary depending on the biomass used as feedstock and the production process. This variability can result in heterogeneity among biochar types used in animal nutrition, leading to inconsistent outcomes. The aim of this study was to characterize the metabolomic and functional properties of an aqueous biochar extract from vine pruning waste, in order to predict its potential in vivo effects as a functional feed ingredient. A metabolomic analysis of the biochar extracts was conducted using quadrupole time-f-light (QQTOF) high-performance liquid chromatography tandem mass spectrometry (HPLC MS/MS). Antimicrobial activity against *E. coli* F18+ and *E. coli* F4+ was assessed using standard growth inhibition assays, while quorum sensing in *E. coli* exposed to biochar extracts was evaluated using real-time PCR. Prebiotic activity was assessed by exposing selected *Lactobacillus* strains to the biochar extract, monitoring growth patterns to determine species-specific responses. The metabolomic profile revealed several distinct molecular classes, including multiple peaks for phenolic compounds. The extract significantly inhibited the growth of both *E. coli* pathotypes, reducing growth by 29% and 16% for the F4+ and F18+, respectively (*p* < 0.001). The relative expression of the genes involved in quorum sensing (MotA, FliA for biofilm formation, and FtsE, HflX for cell division) indicated that the observed inhibitory effects likely resulted from interference with flagellar synthesis, motility, and reduced cell division. The biochar extract also showed species-specific prebiotic potential. In conclusion, biochar derived from vine pruning waste represents a valuable feed ingredient with functional properties that may help to reduce antibiotic use in livestock production.

## Simple Summary

Biochar is a carbon-rich material produced by the thermal decomposition of organic matter (biomass) in an oxygen-limited environment, a process known as pyrolysis. It has attracted significant attention due to its potential environmental benefits, such as carbon sequestration and waste management, promoting a circular economy. The in vivo properties of biochar depend on its physical characteristics, which are influenced by the biomass used as feedstock and the production process. This results in variation among biochar samples. The goal of this study was to assess the functional properties of biochar produced from vine pruning waste, focusing on its chemical composition, antimicrobial activity against *Escherichia coli*, and prebiotic activity for the *Lactobacillus* species. The extract was rich in components with antimicrobial and antioxidant properties, showing significant inhibitory activity against *E. coli*, a causative agent of enteric disease in young pigs. This inhibitory activity led to a downregulation of genes involved in quorum sensing (biofilm formation and cellular division). Additionally, the biochar extract demonstrated potential species-specific prebiotic activity. Biochar from vine pruning biomass thus represents a valuable feed ingredient with functional properties that may reduce antibiotic use in livestock.

## 1. Introduction

Biochar is a carbon-rich, porous material derived from the thermal decomposition of organic biomass at high temperatures (350 to 1000 °C) in low-oxygen conditions [1]. Traditionally, biochar has attracted interest for its applications in agriculture and environmental management, particularly in soil management and carbon sequestration. Biochar has also gained interest in applications such as environmental remediation, construction materials, horticulture, and climate change mitigation [2,3].

Since 2011, in animal nutrition, biochar has been included in the Catalogue of Feed Materials as a vegetable carbon, a product obtained by carbonizing organic plant material (Reg. EU 2022/1104). It has also attracted attention due to its bioactivity when administered in feed. 

Biochar’s unique properties, such as high porosity and extensive surface area, enable it to adsorb harmful substances, including mycotoxins, plant toxins, pathogens, pesticides, and metabolic toxins [4]. Adsorption can occur physically, where mycotoxins are trapped in the biochar’s pores through van der Waals forces and hydrogen bonding, or chemically, where functional groups (e.g., carboxyl, hydroxyl, and phenolic) interact with mycotoxins. These qualities have led to biochar being recognized globally for its benefits in animal nutrition and environmental management, with ongoing research exploring its potential applications. Several studies have shown that incorporating biochar into animal feed at 1–3% of dry matter can enhance various aspects of animal health and performance, including weight gain, feed efficiency, and product quality [5,6,7]. Dietary biochar has shown benefits across multiple species, including ruminants, swine, poultry, and fish [8]. In growing pigs, 2% biochar in the diet improved nutrient digestibility, particularly in terms of crude fiber and crude protein, although no significant differences in performance were observed [9]. 

Biochar has been shown to enhance growth performance in poultry, improve blood characteristics, reduce fecal microbial shedding, and enhance digestive health and feed efficiency [10]. Additionally, biochar appears to reduce greenhouse gas emissions, particularly methane from ruminants [11]. 

Although studies have demonstrated biochar’s efficacy as a functional ingredient, the fact that is heterogeneous affects how it is applied and its overall effectiveness. 

Since biochar’s properties play a key role in its effects on animals and in vitro fermentation, characterizing biochars is necessary for comparisons across studies. Biochar enhances growth rates and/or feed conversion efficiency in pigs, poultry, and ruminants. Improved feed efficiency in pigs may be associated with the increased villus height induced by biochar. However, not all studies on biochar have reported positive effects on nutrient digestibility or performance outcomes.

The physical characteristics of biochar depend on factors related to the production process and feedstock type [12]. These factors influence biochar properties, including porosity, surface area, particle size, and density [13]. Higher temperatures tend to produce biochar with a greater porosity and surface area. Temperatures of 350 to 500 °C yield biochar with a higher volatile matter content and lower surface area, while temperatures above 500 °C produce biochar with reduced volatile matter, increased fixed carbon, and an enhanced surface area. The heating rate and residence time also affect biochar’s internal structure. This variability makes biochar versatile but challenges standardization across fields. Therefore, a preliminary in vitro evaluation of biochar’s characteristics is essential for optimizing its use.

This study focuses on characterizing the functional properties of biochar derived from vine pruning residues to determine its suitability as a livestock feed additive.

## 2. Materials and Methods

### 2.1. Biochar Samples

A sample of 100 g of vine biochar (VB) was purchased from Romagna Carbone (Bagnocavallo, Ravenna, Italy). It was produced via the slow pyrolysis of vine pruning waste at temperatures ranging from 450 °C to 500 °C in accordance with the protocol of a proprietary process.

### 2.2. Chemical Composition and Mineral Contents

Chemical analyses were conducted in triplicate, following the Official Methods of Analysis by AOAC International [14]. The contents of ash, crude fiber (CF), crude protein (CP), and ether extract (EE) were measured. The dry matter (DM) was determined by placing the samples in a forced-air oven at 65 °C for 24 h (AOAC method 930.15). The ash content was measured by heating the samples in a muffle furnace at 550 °C for three hours (method 942.05). CF was analyzed using the filter bag method (AOCS method Ba 6a-05), while CP was assessed using the Kjeldahl method (AOAC method 2001.11). The EE content was measured using ether extraction in the Soxhlet system (DM 21/12/1998).

The mineral composition was evaluated using calibration curves for each target element (Na, Mg, Al, K, Ca, Cr, Mn, Fe, Co, Ni, Cu, Zn, As, Se, Mo, Cd, Pb, P). The samples (0.3 g) were dried and digested in 10 mL of 65% HNO_3_ in Teflon tubes, then heated to 120 °C over 10 min, and kept at this temperature for another 10 min. After mineralization, the samples were cooled for 20 min and transferred to polypropylene test tubes. The diluted mineralized sample was prepared in a 1:100 ratio using 0.3M HNO_3_ in Milli-Q water. The elemental concentrations were measured using ICP-MS (BRUKER Aurora-M90 ICP-MS). The nebulization performance was verified by adding a 2 mg/L standard solution (72Ge, 89Y, 159Tb) to the samples, resulting in a final concentration of 20 μg/L. A collision reaction interface (CRI) with an H_2_ flow of 80 mL/min through the skimmer cone was used to eliminate polyatomic interference.

### 2.3. Biochar Aqueous Extracts

Vine biochar (VB) was extracted using an eco-friendly green chemical method, adapted from Lou et al. [15] with minor modifications. The biochar was manually homogenized using a mortar and pestle. Ten grams of biochar were suspended in 200 mL of demineralized water and heated in a water bath at 90 °C for four hours. The mixtures were then subjected to rotary shaking at 180 rpm at room temperature (25 °C) for 24 h. The liquid phase was separated from the biochar using centrifugation at 4 °C for 20 min and filter-sterilized with a 0.22 μm filter. The pH of the aqueous extract was measured using a pH meter (ThermoFisher Scientific, Waltham, MA, USA) and stored at −20 °C until further analysis.

### 2.4. QTOF HPLC MS/MS Metabolomic Characterization

The qualitative analysis of the biochar aqueous extract was performed using Q-TOF HPLC MS/MS (Sciex, Framingham, MA, USA). The separation was conducted on a Synergy Hydro-RP 80Å LC column (250 mm, 4.6 mm, 4 µm; Phenomenex, LaneCove, Australia) with a binary mobile phase: A (H_2_O with 1% formic acid) and B (acetonitrile with 0.1% formic acid). The gradient elution program was set at a flow rate of 0.4 mL/min with the following steps: from 0 to 5 min, 0.5% A and 95.5% B; from 5 to 25 min, 75% A and 25% B; from 25 to 31 min, 5% A and 95% B; from 31 to 35 min, 95% A and 5% B. The injection volume was 10 μL. The MS/MS analysis was performed in both the positive and negative ion modes. The raw data were analyzed using two libraries: “metabolites” and “natural compounds” (Sciex OS version 3.1, Framingham, MA, USA).

### 2.5. Evaluation of Total Polyphenol Content 

The total polyphenol content (TPC) was measured using the Folin–Ciocalteu colorimetric method, based on the procedure by Makkar et al. [16]. Two mL of the Folin–Ciocalteu (FC) reagent was added to 2 mL of the biochar aqueous extract. After three minutes, 750 µL of anhydrous sodium carbonate solution (7.5%, *w*/*v*) was added, and the mixture was diluted to 10 mL with distilled water. After one hour, the absorbance was recorded at 765 nm. Calibration curves were created using tannic acid as a standard, with concentrations ranging from 0–240 μg/mL. Each sample and standard calibration point was measured in triplicate. The TPC was expressed as the tannic acid equivalent (TAE) in μg/g biochar.

### 2.6. ABTS Radical Scavenging Assay

The antioxidant capacity (AOX) of the biochar aqueous extract was determined following Ilyasov et al. [17]. A Trolox stock solution (2.5 mM in distilled water) was used to construct the standard curve. A solution of 2,2′-azinobis (3-ethylbenzothiazolin-6-sulphonic acid (ABTS) (7 mM) was prepared with 140 mM potassium persulphate in distilled water and left to react in the dark for 12 h to form the ABTS^+^ solution. For the AOX capacity assay, the ABTS^+^ solution was diluted with phosphate-buffered saline (PBS) at pH 7.4 to reach an absorbance of 0.706 ± 0.01 at 734 nm. Ten µL of the sample or Trolox standard were mixed with 1 mL of ABTS^+^ solution and incubated for 15 min in the dark at room temperature. The absorbance was then measured at 734 nm using a spectrophotometer. The percentage inhibition of absorbance at 734 nm was calculated and plotted against the Trolox standard curve. The AOX results were expressed as µmol Trolox equivalents (TE)/g extract.

### 2.7. E. coli Growth Inhibitory Activity

The biochar extract was tested for its inhibitory effect on *E. coli* growth, specifically on two enterotoxigenic *E. coli* (ETEC) strains with F4 (F4+) and F18 (F18+) adhesive fimbriae. These strains were obtained from the Department of Veterinary Medicine and Animal Science at the University of Milan (Italy). *E. coli* strains, grown overnight, were inoculated into tubes containing 15 mL of Luria Bertani (LB) medium supplemented with biochar extract at concentrations of 0, 25, 50, and 100 µL/mL. The tubes were incubated aerobically at 37 °C with shaking at 180 rpm. 

Bacterial growth was monitored by measuring the optical density at 600 nm (OD_600_) of each culture every 60 min using a spectrophotometer (UV/VIS Lambda 365, PerkinElmer, Waltham, MA, USA). Bacteria-free tubes with corresponding biochar extract concentrations served as blank controls. All the optical density readings were converted to cell counts (CFU/mL) using a calibration curve.

### 2.8. RNA Extraction and Real-Time PCR

Total RNA was extracted from *E. coli* F4+ and F18+ after three hours of co-culture with biochar extract at the highest tested dose (100 µL/mL) for growth inhibitory activity. The RNA extraction used the RNA Basic KIT FastGene (Nippon Genetics Europe, Duren, Germany). The RNA integrity and concentration were assessed using a Nanodrop spectrophotometer (ThermoFisher Scientific, Waltham, MA, USA). The cDNA synthesis was performed using the iScript cDNA Synthesis Kit (BioRAD, Hercules, CA, USA), following the manufacturer’s instructions. The quantitative real-time PCR (qRT-PCR) was conducted using the SsoAdvanced Universal SYBR Green Mix (BioRad, Hercules, CA, USA). The qRT-PCR protocol included an initial denaturation at 95 °C for five minutes, followed by 40 cycles of 95 °C for 15 s and 60 °C for 30 s. A melting curve was generated from 65 °C to 95 °C, with increments of 0.5 °C every ten seconds. The primers used are listed in Table 1. The relative expression was calculated using the 2^−ΔΔCT^ method with the GapA gene as an internal control [18].

### 2.9. The Effects on Growth of Biochar Extract on Lactiplantibacillus Plantarum and Limosilactobacillus Reuteri

The biochar extract was tested on the growth of two probiotic strains (*L. plantarum* and *L. reuteri*) obtained from the private collection of Biotecnologie BT (Todi, Italy) and previously evaluated in vivo [20]. Bacteria grown in Man Rogosa Sharpe (MRS) broth for 12 h at 30 °C without shaking were used as inoculants for all the experiments. Overnight cultures were inoculated into tubes containing 15 mL of MRS medium supplemented with biochar extract at concentrations of 0, 50, and 100 μL/mL. The tubes were then incubated at 30 °C. Bacterial growth was measured by recording the optical density at 600 nm (OD_600_) every two hours using a spectrophotometer (UV/VIS Lambda 365, PerkinElmer, Waltham, MA, USA).

### 2.10. Statistical Analysis

Data are presented as mean ± standard deviation (*n* = 3). The statistical analysis was conducted using GraphPad Prism (v. 9.0.0, 2020). Differences between treatments were analyzed using two-way analysis of variance (ANOVA). A single asterisk (*) indicates a significant difference at *p* ≤ 0.05, two asterisks indicate *p* ≤ 0.01, and three asterisks indicate *p* ≤ 0.001.

## 3. Results

### 3.1. Chemical Composition and Mineral Content of Vine Biochar

The DM content of VB biochar was 95.08 ± 0.06%. The chemical composition was characterized by the following percentages of macronutrients expressed as fed: 15.00 ± 0.23% ash, 4.10 ± 1.28% CF, CP ≤ 1.00%, and EE not detectable. Elemental concentrations, which were evaluated using ICP-MS, revealed a varied profile of both macro- and microminerals (Table 2), including key nutrients as well as traces of contaminants such as heavy metals.

### 3.2. Characterization of the Bioactive Compounds in the Biochar Extract

The metabolomic analysis of the VB extract, conducted using QTOF HPLC MS/MS, produced a chromatogram showing various molecular classes, indicating molecular richness. Multiple peaks corresponded to phenolic compounds and bioactive molecules (Table 3), including hydroxybenzoic acid, gallic acid, cresotinic acid, and hydroxytyrosol. The VB extract also contained carboxylic acids known for their antioxidant and antimicrobial properties, including mono- and dicarboxylic acids as well as aromatic acids.

The mono- and dicarboxylic acids identified included valproic, caprylic, nicotinic, and picolinic acids. The dicarboxylic acids included azelaic acid and phthalic acid. The aromatic acids, where the carboxyl group is attached to a benzene ring, included hydrocinnamic acid. The extract also contained methylcoumarin.

### 3.3. ABTS Radical Scavenging Assay and Total Polyphenol Content

The pH of the VB extract was 9.48 ± 0.15. In line with the metabolomic characterization, the water extract showed an antioxidant (AOX) activity of 108.90 ± 1.96 µmol/g of Trolox equivalents (TEs) and a total polyphenol content of 434.33 ± 3.21 µg/g.

### 3.4. Growth Inhibitory Activity

**Figure 1:**
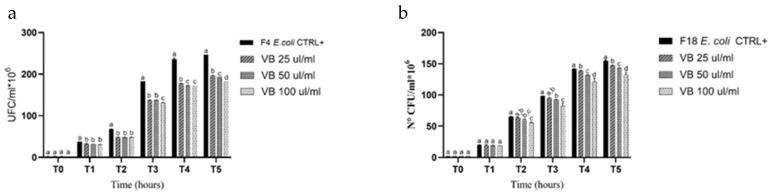
Assessment of growth inhibition by the VB extract against *E. coli*. (**a**) Growth inhibition of *E. coli* F4+. (**b**) Growth inhibition of *E. coli* F18+. Data are shown as means and standard deviations. Different superscript letters indicate significant differences at *p* < 0.05 among different concentrations within the same time point.

The growth of the *E*. *coli* F4+ and F18+ strains was tested with several concentrations (0, 25, 50, and 100 µL/mL) of the VB extract. In the control group (without the VB extract), *E*. *coli* F4+ and F18+ strains showed no lag phase and entered the logarithmic growth phase after two hours (Figure 1). Compared with the negative control, the VB extract exhibited an inhibitory effect against both pathotypes, with growth inhibition observed at all the concentrations.

For *E. coli* F4+, inhibition began after one hour of exposure, while for *E. coli* F18+, inhibition started after two hours and continued for the following three hours. The maximum inhibition rate for *E. coli* F4+ at the highest concentration (100 µL/mL) was 29% at two hours (T2) of exposure, decreasing slightly to 26.5% by the end of the experiment (T5) with a reduction of 2.5%. For *E. coli* F18+, the peak inhibition rate was 16%, occurring after two hours of incubation with the extract (T3) (Figure 1). Under the experimental conditions used, *E. coli* F4+ was found to be more sensitive to the VB extract than *E. coli* F18+ (Figure 1).

### 3.5. Relative Expression of Genes Involved in Quorum Sensing (QS) in E. coli in the Presence of the VB Extract

To understand how the VB extract affects the *E. coli* growth at the molecular level, qRT-PCR was conducted on four genes involved in both the QS regulation of biofilm formation (FliA and MotA) and cell division and proliferation (FtsE and HflX).

**Figure 2:**
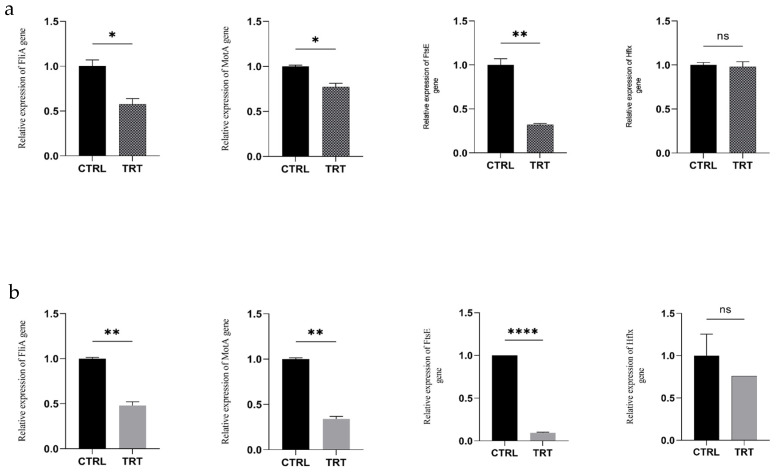
Relative expression of FliA, MotA, FtsE, and Hflx genes. (**a**) Relative expression for *E. coli* F18+ at 3 h of coculture with 100 μL/mL of the VB extract; (**b**) Relative expression for *E. coli* F4+ at 3 h of coculture with 100 μL/mL of the VB extract. * indicates *p* ≤ 0.05; ** indicates *p* ≤ 0.01; **** indicates *p* ≤ 0.0001.

The results showed that the VB extract significantly downregulated the expression of the QS genes FliA, MotA, and FtsE in both pathotypes. In contrast, the HflX gene was not significantly differentially expressed (Figure 2a,b).

### 3.6. L. plantarum and L. reuteri Growth in the Presence of the CB Biochar Hot Water Extract

**Figure 3:**
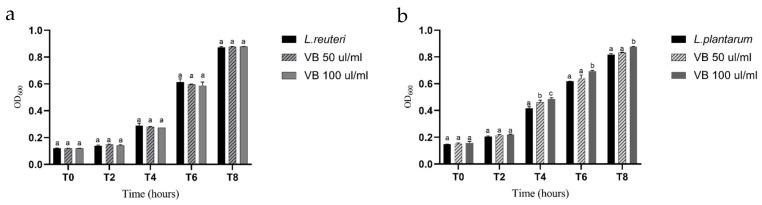
(**a**) *L. reuteri* growth in the presence of 0, 50, and 100 μL/mL of VB biochar over time; (**b**) *L. plantarum* growth in the presence of 0, 50, and 100 μL/mL of VB biochar over time. Different superscript letters indicate significant differences at *p* < 0.05 among different concentrations within the same time point.

Neither of the tested concentrations (50 and 100 µL/mL) exhibited any inhibitory effects on *L. plantarum* and *L. reuteri*. The growth curve of *L. reuteri* in the presence of the CB extract was similar to the negative control (without the VB extract). Interestingly, *L. plantarum* showed a significant increase in growth (*p* ≤ 0.05) from 4 to 6 h of contact with the VB extract (Figure 3).

## 4. Discussion

Biochar is a highly variable carbon-rich product derived from the thermal decomposition of organic materials in an oxygen-limited environment. Its popularity has increased due to its ability to reduce the environmental impact of biomass and contribute to carbon sequestration. This process helps to store carbon in the soil for long periods, thereby reducing greenhouse gas emissions. 

Carbon sequestration is particularly relevant to the carbon credit market, which is expected to grow as efforts to achieve net-zero emissions intensify. The physicochemical properties of biochar are critical for determining its bioactivity and effectiveness in vivo, especially when used as animal feed. These properties, along with the quality, yield, and potential toxicity of biochar, are influenced by various factors, including the type of feedstock, the production technology, and the specific process conditions. Consequently, biochar products are highly heterogeneous, affecting their performance and bioactivity.

Given the wide-ranging applications of biochar, it is essential to understand its specific functional properties, especially when used as a feed ingredient. Our study focused on biochar produced from vine prunings, offering an opportunity to convert this agricultural by-product into a functional and sustainable feed ingredient. This approach not only reduces agro-residual waste but also contributes to resource reusability, thus promoting sustainability in agricultural practices. Vineyards can produce approximately 1 to 10 tons of pruning waste per hectare per year, with winter pruning wood typically destroyed by burning or crushing on the ground [21].

The chemical characterization of vine biochar showed a dry matter (DM) content of 95.08 ± 0.06%, indicating a highly concentrated product with minimal moisture due to the high temperatures used during pyrolysis [22]. This low moisture content makes it suitable for animal feeds, as it appears not to significantly alter the moisture content of the feed, which must remain below 12% [23]. The low water content also reduces the risk of bacterial contamination and fungal growth, making the biochar easier to store safely. 

During pyrolysis, organic materials are heated in the absence of oxygen, thereby decomposing organic volatile compounds and water. Higher temperatures drive off more volatiles, leaving a product rich in carbon and other stable compounds [24]. 

The absence of detectable ether extract and the low protein content are consistent with high-temperature pyrolysis, which typically degrades these components [25]. The chemical composition of the VB, particularly its ash content (15.00 ± 0.23%), reflects the presence of significant amounts of minerals. This likely results from the high pyrolysis temperatures, which degrade organic matter, concentrate minerals, and are influenced by the intrinsic characteristics of the original biomass [26].

Minerals are essential in animal nutrition and are classified as essential, non-essential, or contaminants, such as heavy metals. An in-depth evaluation of the mineral profile is crucial to ensure that the product meets nutritional requirements, while minimizing potential contaminants (Dir. 2002/32/EC).

In this study, an elemental analysis using ICP-MS revealed a varied profile of both macro- and microminerals, including essential nutrients. Importantly, the concentrations of heavy metals, such as arsenic (As), lead (Pb), and cadmium (Cd), were below the threshold levels for biochar as a feed ingredient. This ensures that VB biochar is safe in animal diets without posing risks of toxicity from heavy metals.

The vine biochar analyzed in this study contained high levels of calcium (Ca), potassium (K), magnesium (Mg), and phosphorus (P), as these minerals are less likely to volatilize at lower pyrolysis temperatures (350–500 °C) and are abundant in the original biomass. In animal nutrition, these minerals are classified as macroelements, with the required levels significantly higher than those of the trace elements. Therefore, it is important to define the macroelement content before introducing the biochar into animal feed to ensure compliance with the mineral limits imposed by regulations.

In biochar derived from vine pruning residues, particular attention should be given to copper (Cu). Our results indicate considerable levels of copper, though these are still below the regulatory limits for commercial raw materials. This may be related to its extensive use in agriculture, particularly in vineyards, where copper-based fungicides are applied, leaving copper residues on the biomass after pruning [27]. Although copper is an essential nutrient in animal physiology, playing a crucial role in enzyme function, immune response, and iron metabolism, its use has recently raised concerns [28]. Copper can contribute to eutrophication and it is involved in the co-selection of antibiotic-resistant bacteria [29]. For these reasons, the European Union sets maximum copper levels in animal feed for certain categories to ensure safety and prevent environmental contamination [30]. Therefore, if VB biochar is used in the preparation of complete feed for animals, it should be considered a potential copper source to ensure compliance with the established European maximum copper level.

Vine pruning biomass is rich in polyphenols, which are organic compounds characterized by multiple phenol units (i.e., aromatic rings with hydroxyl groups attached). The metabolomic profile in this study revealed that the VB extract is rich in small polyphenols containing 4–8 carbon atoms, likely due to the pyrolysis process. During pyrolysis, polyphenols undergo thermal degradation and transformation. Thermal energy breaks the chemical bonds within polyphenolic structures, forming smaller molecules. Functional groups, such as hydroxyl (-OH), carboxyl (-COOH), and methoxy (-OCH_3_), attached to polyphenolic rings, are often lost or transformed during pyrolysis [30].

Among the polyphenols, the VB extract contains hydroxybenzoic acid, gallic acid, cresotinic acid, and hydroxytyrosol, all of which have antioxidant and antimicrobial properties. These compounds scavenge free radicals and interfere with microbial enzymes and proteins, inhibiting their function and leading to microbial cell death [31]. The VB extract also contains methylcoumarins, which are coumarin molecules with methyl groups (-CH_3_) at various positions on the coumarin ring structure. Coumarin derivatives, including methylcoumarins, have been studied for their antioxidant and antimicrobial properties [32].

Metabolomic analyses confirm the antioxidant activity of the VB extract. The VB extract has an AOX activity that is perfectly in line with the data on the AOX activity of vine pruning waste of 100–500 μmol TE/gr DW [33]. This property makes biochar from vine waste suitable for various applications, including as a natural source of antioxidants and in animal feed. Antioxidant ingredients could help in reducing oxidative damage in animals, contributing to overall health and performance. They are among the most studied alternatives to antibiotics in animal nutrition, and are integral to agroecology and sustainable development in terms of the One Health approach [34].

VB biochar inhibits the growth of *Escherichia coli* strains characterized by fimbrial virulence factors (specifically F4 and F18) and verocytotoxins. *Escherichia coli* strains were chosen as model organisms because they are primarily responsible for severe enteric and systemic diseases in pig farming, particularly in weaned pigs. The VB extract used in this study significantly inhibited both *E. coli* strains, likely due to the presence of functional compounds with known antimicrobial properties identified in the extracts. Additionally, the VB extract showed inhibitory effects against *E. coli* F4+ and F18+ strains, particularly at higher concentrations. The earlier inhibition observed in *E. coli* F4+ (starting at 1 h) compared with *E. coli* F18+ (starting at 2 h) suggests that the extract is more effective against certain *E. coli* pathotypes. The decline in inhibition over time could indicate adaptive bacterial responses or the depletion of active compounds. 

Nevertheless, the extract’s ability to inhibit bacterial growth supports its potential role in improving feed safety by reducing pathogenic bacteria. Recent studies, such as Wang et al. [35], have shown that phenolic compounds like 4-hydroxybenzoic acid can affect bacterial growth and morphology by inhibiting those enzymes involved in quorum sensing (QS) signal synthesis or degradation. QS is a communication mechanism that bacteria use to coordinate behavior, including virulence factor production. 

Targeting QS offers an effective strategy for inhibiting virulence without killing the bacteria, thus reducing the risk of resistance development. To better understand the antibacterial mechanism of VB biochar, we investigated the expression of the genes involved in QS signaling and *E. coli* pathogenicity, focusing on its potential to reduce virulence. Biofilm formation in *E. coli* is regulated by a complex network of genes and pathways that coordinate bacterial activity based on cell density. In *E. coli*, the FliA gene encodes sigma factors (σ70 or σ28) that regulate flagellar biosynthesis, including flagellin (FliC). Additionally, FliA controls the transcription of most motility-related genes, including MotA, which is essential for the bacterial flagellar motor. In contrast, the FtsE gene plays a role in cell division and septation, encoding a membrane protein that, together with FtsX, forms the FtsEX complex that regulates the early stages of cell division.

We found that the expression of QS-related genes (FliA, MotA, and FtsE) was downregulated in the presence of the VB extract. This indicates that bioactive components in the biochar may disrupt bacterial communication and biofilm formation. This effect could potentially reduce the virulence and persistence of pathogenic bacteria in the gut, supporting the use of biochar as a functional feed additive. 

Previous studies have reported similar downregulation patterns in QS genes in response to various treatments. For example, certain plant extracts or antimicrobial agents downregulate QS genes, interfering with bacterial motility and biofilm formation [36]. The downregulation of FliA and MotA, which are involved in bacterial motility and chemotaxis, leads to the reduced expression of flagellar components and impaired flagellar motor function, ultimately decreasing motility. Since flagellar motility in pathogenic bacteria is often linked to virulence, the downregulation of FliA and MotA could weaken the bacteria’s ability to invade host tissues or evade immune responses. 

Reduced motility also impairs biofilm formation, which is essential for bacterial persistence and infection in various environments. Furthermore, the downregulation of the FtsE gene, which is involved in the divisome complex responsible for cell division, impacts bacterial growth. Reduced FtsE expression can slow growth rates due to inefficient cell division. Phenolic compounds in VB biochar may influence the expression of genes such as FtsE by affecting transcriptional regulators or altering bacterial stress responses. Many phenolic compounds are known for their antibacterial activity, which may involve mechanisms that disrupt cell division.

In this study, we also explored how water-soluble compounds in VB biochar influence the growth of *L. plantarum* and *L. reuteri*, strains isolated from weaned piglets and previously characterized in vitro and in vivo [20,37]. We found no inhibitory effects on beneficial bacteria, such as *L. plantarum* and *L. reuteri*, suggesting that VB biochar may selectively inhibit pathogenic bacteria while supporting the growth of beneficial microbes. We also observed how *L. plantarum* growth was stimulated in the presence of the VB extract, which highlights the prebiotic effect of biochar, promoting a healthy balance in the gut microbiota.

Our findings indicate that VB biochar could exert selective prebiotic activity, as only *L. plantarum* showed enhanced growth among the two strains tested. This result aligns with previous studies showing that biochar can either stimulate or inhibit bacterial growth, even within the same species. For instance, Fan Yang et al. [38] investigated the effects of biochar on various bacterial strains and reported that washed biochar could either increase or decrease colony-forming units (CFUs), emphasizing the role of water-soluble compounds in fresh biochar in modulating bacterial growth. Their results confirmed that the effect is highly strain-dependent. Other studies suggest that biochar can positively influence the gut microbiota and bacterial metabolism. Han et al. [39] demonstrated that administering rice straw biochar to rats significantly altered cecal bacterial communities by increasing beneficial *Firmicutes* and reducing opportunistic pathogens.

However, exactly how biochar affects the growth of different bacterial strains remains unclear, and requires further investigation. Our in vitro study needs to be confirmed by in vivo studies, but translating the effects that we observed to real-world conditions is challenging. 

A key factor is to understand the most effective percentage of biochar in the feed in order to optimize the positive effects and minimize any negative effects. High inclusion percentages may negatively impact digestibility due to the material’s porous and absorbent nature. This study also revealed that biochar can selectively modulate microorganism development. This aspect is still poorly understood, and again, further investigation is necessary, both to understand the mechanism of action (strain-dependent effects) and to explore its broader influence, such as its potential to modulate the intestinal microbiota.

## 5. Conclusions

We investigated the functional properties of an aqueous extract of biochar from vine pruning waste, highlighting its potential as a sustainable feed additive. VB biochar selectively promoted the growth of beneficial bacteria, such as *L. plantarum*, while inhibiting pathogenic *Escherichia coli* strains associated with fimbrial virulence factors (F4+ and F18+). The bioactive compounds identified in the VB extract, including small polyphenols and methylcoumarins, demonstrated both antioxidant and antimicrobial activities, disrupting bacterial enzymes, proteins, and membranes. The extract’s antioxidant capacity aligns with the previously reported values for vine pruning waste, supporting its potential to reduce oxidative stress in animal feed.

VB biochar has the potential to improve gut health, reduce pathogenic load, and serve as a natural alternative to antibiotics, thereby aligning with sustainable agriculture and the One Health approach. Due to its inherent properties, biochar can selectively influence microorganism development. Further research is needed to explore its strain-specific effects and optimize its application.

## Figures and Tables

**Table 1:** List of primers used in this study for qRT PCR.

Target	Nucleotide Sequence	Acc. N°	Size	Ref.
GapA	FW-GAAATGGGACGAAGTTGGTG Rv-AACCACTTTCTTCGCACCAG	NP_416293	104 bp	[19]
FliA	FW-GCTGGCTGTTATTGGTGTCG Rv-CAACTGGAGCAGGAACTTGG	NP_416432	112 bp	[19]
MotA	FW-CTTCCTCGGTTGTCGTCTGT Rv-CTATCGCCGTTGAGTTTGGT	NP_416404	120 bp	[19]
FtsE	FW-AAAGTACCCTCCTGAAGCTGATCTGTG Rv-GCGTGATGTCATGGCCGCTAAAC	NP_417920	81 bp	[19]
HflX	FW-TGTAGGTGAAGGTAAAGCAG Rv-CACGACACTCGCACAAACGC	NP_418594	128 bp	[19]

**Table 2:** Mineral content of VB biochar using ICP-MS. The results are expressed as mean ± SD (*n* = 3).

Mineral	Content (g kg^−1^)	Mineral	Content (mg kg^−1^)	Mineral	Content (mg kg^−1^)
Na	0.76 ± 0.48	Cr	5.53 ± 3.21	Zn	134.30 ± 134.65
Mg	6.35 ± 4.05	Mn	165.77 ± 109.23	As	1.44 ± 0.32
Al	1.80 ± 1.18	Fe	1.53 ± 0.85	Se	0.04 ± 0.00
P	3.89 ± 2.68	Co	0.40 ± 0.55	Mo	n.r
K	19.82 ± 13.21	Ni	5.03 ± 3.50	Cd	0.05 ± 0.01
Ca	9.21 ± 7.06	Cu	291.23 ± 190.43	Pb	3.26 ± 2.15

**Table 3:** Metabolomic profile of the VB extract.

Component	Formula	Area	Retention Time	Adduct
4-Hydroxybenzoic acid	C_7_H_6_O_3_	108484	8.34	[M − H]^−^
Azelaic acid	C_9_H_16_O_4_	1830729	8.03	[M − H]^−^
Caprylic acid	C_8_H_16_O_2_	43483	8.03	[M − H]^−^
6-Methylcoumarin	C_10_H_8_O_2_	20094	8.27	[M − H]^+^
7-Hydroxycoumarin	C_10_H_8_O_3_	2716974	7.09	[M − H]^+^
Nicotinic acid	C_6_H_5_NO_2_	97166	1.64	[M − H]^+^
Gallic acid	C_7_H_6_O_5_	125658	4.54	[M − H]^+^
Cresotinic acid	C_8_H_8_O_3_	382887	7.12	[M − H]^−^
Hydrocinnamic acid	C_9_H_10_O_2_	29782	6.02	[M − H]^+^
Phthalic acid	C_8_H_6_O_4_	254600	5.62	[M − H]^+^
Picolinic acid	C_6_H_5_NO_2_	97166	1.64	[M − H]^+^
Pyroglutamic acid	C_5_H_7_NO_3_	2002	1.66	[M − H]^+^
Citraconic acid	C_5_H_6_O_4_	1455371	1.73	[M − H]^−^
Hydroxytyrosol	C_8_H_10_O_3_	330231	1.86	[M − H]^−^

## Data Availability

The authors confirm that the data supporting the findings of this study are available within the article.
